# Expression Profile of the IL‐17 Receptor Family on Peripheral B Lymphocytes in Rheumatoid Arthritis

**DOI:** 10.1155/jimr/5266857

**Published:** 2026-10-23

**Authors:** Coronado González Christian Fernando, Sifuentes Franco Sonia, Hernández Bello Jorge, Muñoz Valle José Francisco, Graciano Machuca Omar, López Pérez Iván, Morales Núñez José Javier

**Affiliations:** ^1^ Programa Educativo Médico Cirujano y Partero Centro Universitario de Ciencias de la Salud (CUCS) Universidad de Guadalajara Guadalajara Jalisco Mexico udg.mx; ^2^ Laboratorio de Ciencias Clínicas Departamento de Ciencias de la Salud Centro Universitario de los Valles Universidad de Guadalajara Ameca Jalisco Mexico udg.mx; ^3^ Instituto de Investigación en Ciencias Biomédicas (IICB) Departamento de Biología Molecular y Genómica Centro Universitario de Ciencias de la Salud Universidad de Guadalajara Guadalajara Jalisco Mexico udg.mx; ^4^ Departamento de Ciencias de la Salud Enfermedad como Proceso Individual Centro Universitario de Tonalá Universidad de Guadalajara Tonalá Mexico udg.mx; ^5^ Departamento de Morfología Centro Universitario de Ciencias de la Salud Universidad de Guadalajara Guadalajara Jalisco Mexico udg.mx

**Keywords:** B cells, IL-17 receptor, rheumatoid arthritis

## Abstract

B cells are key contributors to the immunopathogenesis of rheumatoid arthritis (RA), yet the expression profile of interleukin‐17 (IL‐17) receptors on human B cells remains incompletely defined. We analyzed the surface expression of IL‐17RA–IL‐17RE on peripheral CD19^+^ B cells from 42 patients with RA and 20 healthy controls (HC) using flow cytometry. Receptor‐positive cell frequency and mean fluorescence intensity (MFI) were evaluated, and disease activity was assessed using the Disease Activity Score in 28 joints based on erythrocyte sedimentation rate (DAS28‐ESR). The frequency of receptor‐positive B cells differed significantly among IL‐17 receptor (IL‐17R) family members in both RA and HC, with IL‐17RD showing the highest proportion of positive cells, followed by IL‐17RA, whereas IL‐17RC was less frequent and IL‐17RB and IL‐17RE were rarely detected. The proportion of IL‐17RA^+^ B cells was lower in RA than in HC and remained statistically significant after correction for multiple comparisons (Holm‐adjusted *p* = 0.0126). A nominally lower frequency of IL‐17RC^+^ B cells in RA did not remain significant after adjustment, while IL‐17RD frequency was comparable between groups. No between‐group differences in MFI remained significant after multiple‐comparison correction. Receptor expression was not significantly associated with DAS28‐ESR disease activity categories. These findings provide a descriptive characterization of the IL‐17R family on peripheral B cells in RA and identify a lower frequency of IL‐17RA^+^ B cells as the most robust between‐group difference, suggesting alterations in IL‐17R dynamics that may contribute to B‐cell dysregulation and disease pathogenesis.

## 1. Introduction

Systemic immunological dysfunction, progressive joint deterioration, and persistent inflammation of the synovial membrane are the hallmarks of rheumatoid arthritis (RA), a chronic autoimmune disorder [1–3]. Besides its conventional correlation with the production of autoantibodies, RA is now understood as a condition resulting from intricate interactions between innate and adaptive immune cells, with B cells playing a key role [4–6]. It has been noted that they are involved in the perpetuation of persistent inflammation and tissue damage through the production of the rheumatoid factor (RF) and antibodies against citrullinated proteins (ACPAs), as well as their participation in antigen presentation, cytokine production, and T‐cell response modulation [4, 5, 7].

The interleukin‐17 (IL‐17) axis has been identified as a major contributor to inflammation aggravation within the articular milieu, along with the cytokine pathways linked to RA [8, 9]. IL‐17 has historically been linked to T helper 17 (Th17) cells, and its biological effects are mediated through a range of receptors (IL‐17RA to IL‐17RE) that have different structural characteristics, tissue distributions, and signaling capacities [10–12]. Numerous immune and nonimmune cells have these receptors, which allow IL‐17 to modulate inflammatory responses in several cell types [13, 14]. Nevertheless, little has been researched about the patterns of expression of IL‐17 receptors (IL‐17Rs) on B cells, especially in relation to RA [9].

Increasing evidence indicates that IL‐17 signaling can affect B‐cell biological functions, including differentiation, survival, and activation, thereby influencing humoral immune responses in inflammatory settings [10, 15–17]. Although IL‐17R activation has been shown in experimental studies to regulate B‐cell activity, most of the available evidence comes from in vitro systems or animal models, with no direct assessment in human disease [13, 17]. Significantly, IL‐17A‐ or IL‐17RA‐targeting clinical trials in RA have yielded small or inconsistent therapeutic effects, raising concerns about the complexity of IL‐17 signaling networks and the cell‐specific roles of various receptor subunits in chronic inflammation [18, 19]. These findings emphasize the need to more precisely define the IL‐17R expression pattern in critical immune cell types implicated in the pathophysiology of RA.

In this context, a thorough examination of the IL‐17R family expression on B cells in patients with RA could shed light on phenotypic changes linked to the disease that may have functional and therapeutic significance. Thus, the purpose of this work was to assess the expression profiles of IL‐17R family members in peripheral B cells from RA patients and contrast these patterns with those in healthy individuals. This work aims to advance our understanding of IL‐17‐related pathways in B‐cell‐mediated immunological dysregulation in RA by concentrating on receptor expression rather than cytokine measurement.

## 2. Materials and Methods

### 2.1. Study Design and Participants

A cross‐sectional observational study was conducted including patients diagnosed with RA who attended the Rheumatology Service of the Hospital Civil de Guadalajara “Fray Antonio Alcalde” between August 2024 and June 2025. RA was established by a rheumatologist according to the 2010 ACR/EULAR classification criteria. A control group consisting of clinically healthy individuals without a personal or family history of autoimmune disease was included.

Clinical and sociodemographic information was collected through the medical history at enrollment. Disease activity was assessed using the Disease Activity Score in 28 joints based on erythrocyte sedimentation rate (DAS28‐ESR). According to established cutoff values, patients were classified as remission (DAS28‐ESR <2.6), low disease activity (≥2.6 to ≤3.2), moderate disease activity (>3.2 to ≤5.1), or high disease activity (>5.1) [20]. This classification was used to explore potential associations between disease activity and the expression of IL‐17R family members on B lymphocytes.

Exclusion criteria included overlapping autoimmune diseases, active infection, neoplastic disease, pregnancy, and treatment with biologic drugs or high‐dose glucocorticoids known to directly affect B‐cell populations. The study population was restricted a priori to women because of the higher prevalence of RA among women and to reduce sex‐related biological variability within this study.

A nonprobabilistic convenience sampling method was used. The sample size was estimated using the formula for comparison of two independent means, with a significance level of 0.05 and a power of 90%, assuming a medium effect size, resulting in an estimated sample size of 41.98. This estimation was based on previous observations from our research group showing no differences in the total frequency of circulating B lymphocytes between RA patients and healthy controls (HC) [21]. A total of 42 women with RA and 20 female HC were included.

### 2.2. Sample Collection and Peripheral Blood Mononuclear Cell (PBMC) Isolation

Peripheral venous blood (10 mL) was collected by venipuncture into EDTA‐containing tubes and processed as fresh blood samples. PBMCs were isolated by density‐gradient centrifugation using the Lymphoprep reagent (Axis‐Shield Diagnostics, UK), following the manufacturer’s instructions. Cells were washed in 10 mL of 1× phosphate‐buffered saline (PBS; Caisson Labs) and counted using a Neubauer chamber. Cell viability was assessed by trypan blue exclusion (Sigma–Aldrich Co.) before immunostaining. No fluorescent viability dye was included in the flow cytometry panel; therefore, dead cells were not specifically excluded by a viability gate during cytometric analysis. For each sample, tubes initially containing 3 × 10^5^ cells were prepared for unstained and antibody‐stained conditions.

### 2.3. Flow Cytometry and Immunophenotyping

A six‐color flow cytometry panel was used to identify CD19^+^ B lymphocytes and evaluate the surface expression of IL‐17R family members. The following fluorochrome‐conjugated monoclonal antibodies were used: CD19‐PE (clone 4G7‐2E3, Cat. No. FAB4867P), IL‐17RA‐Alexa Fluor 700 (clone 133617, Cat. No. FAB177N), IL‐17RB‐Alexa Fluor 350 (clone 170220, Cat. No. FAB1207U), IL‐17RC‐Alexa Fluor 488 (clone 309822, Cat. No. FAB22691G), IL‐17RD‐APC (clone 309511, Cat. No. FAB22751A), and IL‐17RE‐Alexa Fluor 594 (clone 934832, Cat. No. FAB8358T) (R&D Systems). Five microliters of each antibody were added to each staining tube according to the manufacturer’s instructions. The final staining volume was 80 μL.

Unstained cells were used to assess baseline fluorescence and autofluorescence. A separate fluorescence‐minus‐one (FMO) control was used for each IL‐17R to define the fluorescence boundary for receptor positivity; events exceeding the corresponding FMO‐defined boundary were considered receptor‐positive. Fluorescence compensation was performed using UltraComp eBeads Plus compensation beads (Thermo Fisher Scientific). Samples were incubated for 30 min at 4°C, washed twice with 1× PBS by centrifugation at 600 × g for 5 min, and finally resuspended in 500 μL PBS before acquisition.

Samples were acquired using an Attune NxT acoustic focusing flow cytometer (Life Technologies) equipped with three excitation lasers: violet 405 nm (50 mW), blue 488 nm (50 mW), and red 637 nm (100 mW). The fluorescence channels used for the panel were VL1 (440/50 nm) for Alexa Fluor 350, VL2 (512/25 nm) for Alexa Fluor 488, VL3 (603/48 nm) for Alexa Fluor 594, BL2 (574/26 nm) for PE, RL1 (660/20 nm) for APC, and RL2 (720/30 nm) for Alexa Fluor 700.

### 2.4. Data Analysis and Statistical Methods

Flow cytometry data were analyzed using FlowJo software (v10.8.1). Lymphocytes were identified according to forward scatter (FSC‐A) and side scatter (SSC‐A) characteristics, followed by singlet discrimination using FSC‐A and FSC‐H. CD19^+^ B lymphocytes were subsequently identified, and IL‐17RA, IL‐17RB, IL‐17RC, IL‐17RD, and IL‐17RE‐positive populations were quantified within the CD19^+^ gate. Positivity thresholds were established using the corresponding FMO control for each receptor. The complete gating strategy for representative RA and HC samples is shown in Figure 1, and the corresponding FMO controls are provided in Figure S1. Event counts for the sequential gating steps and receptor‐positive populations are reported in Table S1.

**Figure 1:**
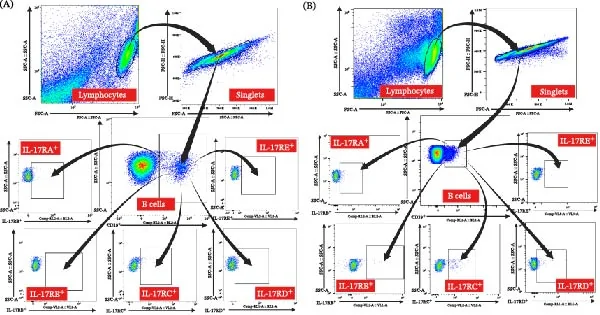
Gating strategy for the identification of IL‐17 receptor‐positive peripheral B cells in patients with rheumatoid arthritis and healthy controls. Lymphocytes were first selected according to forward scatter (FSC‐A) and side scatter (SSC‐A) characteristics, followed by singlet discrimination using FSC‐A and FSC‐H to exclude cell aggregates. CD19^+^ B cells were subsequently identified, and surface expression of IL‐17RA, IL‐17RB, IL‐17RC, IL‐17RD, and IL‐17RE was evaluated within the CD19^+^ B‐cell population. (A) Representative gating strategy from one patient with rheumatoid arthritis (RA; *n* = 42). (B) Representative gating strategy from one healthy control (HC; *n* = 20). Each representative sample corresponds to one independent donor and was stained and acquired once, with no technical replicates. Positivity thresholds for each IL‐17 receptor were established using the corresponding fluorescence‐minus‐one (FMO) control; events exceeding the FMO‐defined fluorescence boundary were considered receptor‐positive. FMO controls are shown in Figure S1, and detailed event counts for the sequential gating steps and receptor‐positive populations are provided in Table S1.

Receptor expression was reported as the percentage of CD19^+^ cells positive for each IL‐17R and, where applicable, as the mean fluorescence intensity (MFI). Because MFI values were obtained using different antibody‐fluorochrome conjugates, they were considered relative fluorescence measurements for each individual conjugate and were not interpreted as calibrated quantitative comparisons of receptor abundance across different IL‐17R family members.

Statistical analyses were conducted using GraphPad Prism (v6.01). Nonnormally distributed continuous variables are presented as median (interquartile range) and categorical variables as *n* (%). Comparisons between two independent groups (RA vs. HC) were performed using the Mann–Whitney *U* test. Nominal *p* values for the six RA‐versus‐HC receptor comparisons were adjusted for multiple comparisons using the Holm method.

Comparisons of IL‐17R family members measured within the same individuals were performed using the Friedman test. When the global Friedman test was significant, pairwise comparisons were conducted using the Wilcoxon signed‐rank test with the Holm adjustment for multiple comparisons.

Comparisons among the four independent DAS28‐ESR disease activity categories were performed using the Kruskal–Wallis test. Because none of the global receptor‐expression comparisons according to disease activity reached statistical significance, no post hoc pairwise comparisons were performed for these analyses. Categorical variables, including treatment frequencies across disease activity categories, were compared using the chi‐square (*χ* ^2^) test. A *p* value < 0.05 was regarded as statistically significant.

### 2.5. Ethical Approval

The study was carried out in compliance with the Declaration of Helsinki 2024 and authorized by the institutional ethics committee of the Centro Universitario de los Valles of the Universidad de Guadalajara (Permission Number CEI/47/2023). Written informed consent was acquired by each subject.

## 3. Results

### 3.1. Clinical and Demographic Characteristics of the Study Population

A total of 42 patients with RA and 20 HC were included. 100% of the participants in both groups were female. The median age was similar between RA patients (50 years [IQR 45–57]) and HC (51.5 years [IQR 43.5–54.3]; *p* = 0.635). RA patients exhibited significantly higher levels of RF (89 U/mL [IQR 61.1–108.7] vs. 0.99 U/mL [IQR 0.99–0.99]; *p* < 0.001), C‐reactive protein (CRP; 2.1 mg/L [IQR 1.7–3.5] vs. 1.5 mg/L [IQR 0.5–3.7]; *p* = 0.038), and ESR (29 mm/h [IQR 24–39.75] vs. 12 mm/h [IQR 11–13]; *p* < 0.001) compared with HC.

Among RA patients, disease duration did not differ significantly across DAS28‐ESR activity categories (*p* = 0.18). The distribution of methotrexate, sulfasalazine, hydroxychloroquine/chloroquine, and paracetamol use was also similar among disease activity groups (*p*  > 0.05), whereas celecoxib use differed across categories (*p* = 0.022) (Table 1).

**Table 1:** Clinical and demographic characteristics of the study population.

Variable			RA (*n* = 42)	Controls (*n* = 20)	*p* value
RA patients versus healthy controls					
Female sex, *n* (%)			42 (100)	20 (100)	—
Age (years)			50 (45–57)	51.5 (43.5–54.3)	0.635
Erythrocyte sedimentation rate (ESR) (mm/h)			29 (24–39.75)	12 (11–13)	<0.001
C‐reactive protein (CRP) (mg/L)			2.1 (1.7–3.5)	1.5 (0.5–3.7)	0.038
Rheumatoid factor (RF) (U/mL)			89 (61.1–108.7)	0.99 (0.99–0.99)	<0.001

**Characteristic**	**Remission (*n* = 10)**	**Low (*n* = 10)**	**Moderate (*n* = 12)**	**High (*n* = 10)**	***p***

Disease duration and treatment according to RA disease activity					
Disease duration (months)	36 (33–78)	84 (42–154)	39 (17–57)	120 (36–240)	0.18
Methotrexate, *n* (%)	10 (100%)	9 (90%)	10 (83.3%)	9 (90%)	0.622
Sulfasalazine, *n* (%)	5 (50%)	7 (70%)	10 (83.3%)	7 (70%)	0.415
Hydroxychloroquine/chloroquine, *n* (%)	2 (20%)	5 (50%)	1 (8.33%)	4 (40%)	0.13
Paracetamol, *n* (%)	1 (10%)	2 (20%)	3 (25%)	4 (40%)	0.46
Celecoxib, *n* (%)	6 (60%)	10 (100%)	12 (100%)	7 (70%)	0.022

*Note*: Values are presented as median (interquartile range) for continuous variables and as *n* (%) for categorical variables. Comparisons between RA and HC were performed using the Mann–Whitney *U* test. Comparisons among disease activity groups were performed using the Kruskal–Wallis test for continuous variables and the chi‐square test for categorical variables.

Abbreviations: CRP, C‐reactive protein; ESR, erythrocyte sedimentation rate; HC, healthy controls; RA, rheumatoid arthritis.

### 3.2. Frequency of Peripheral B Cells

There was no difference in the percentage of circulating CD19^+^ B cells between the groups (*p* = 0.3223) (HC: 6.27% [IQR 4.50–10.21] vs. RA: 7.81% [IQR 6.12–10.85]) (Figure 2A). The MFI of CD19 was similar (Figure 2B), with HC being 2621 (IQR 2361.5–2884.75) and RA being 2536 (IQR 2180.25–2707.25), *p* = 0.2857.

**Figure 2:**
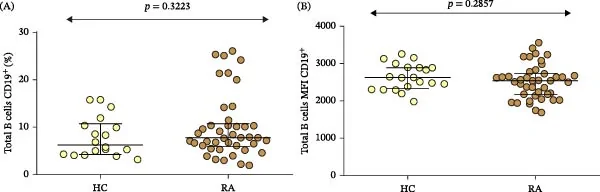
Peripheral B‐cell frequency in patients with rheumatoid arthritis and healthy controls. (A) Percentage of circulating CD19^+^ B lymphocytes in patients with rheumatoid arthritis (RA; *n* = 42) and healthy controls (HC; *n* = 20). (B) Mean fluorescence intensity (MFI) of CD19 expression in RA and HC. Data are presented as medians with interquartile ranges, and each symbol represents one independent donor. Each sample was stained and acquired once, with no technical replicates. Statistical comparisons between RA and HC were performed using the Mann–Whitney *U* test.

### 3.3. Expression Profile of the IL‐17R Family on B Lymphocytes

Within RA samples, the percentage of CD19^+^ B cells expressing IL‐17R family members varied considerably (*p* < 0.0001). IL‐17RD was the most common with a frequency of 14.80% (IQR 12.90–16.40), followed by IL‐17RA with 2.61% (IQR 1.80–3.44) and IL‐17RC with 0.225% (IQR 0.13–0.48). Very low concentrations of IL‐17RB and IL‐17RE were found (0.17% [IQR 0.101–0.295] and 0.14% [IQR 0.09–0.29], respectively) (Figure 3A).

**Figure 3:**
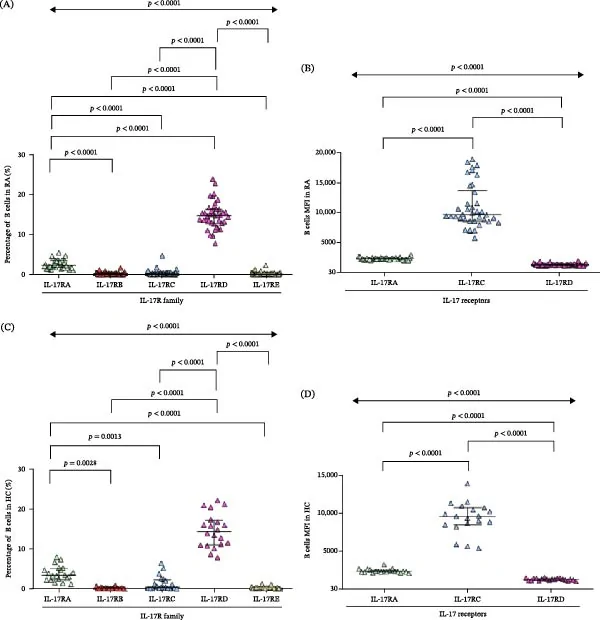
Expression profile of IL‐17 receptor family members on peripheral B lymphocytes. (A) Percentage of CD19^+^ B cells expressing IL‐17RA, IL‐17RB, IL‐17RC, IL‐17RD, and IL‐17RE in patients with rheumatoid arthritis (RA; *n* = 42). (B) Mean fluorescence intensity (MFI) of IL‐17RA, IL‐17RC, and IL‐17RD in patients with RA. (C) Percentage of CD19^+^ B cells expressing IL‐17RA, IL‐17RB, IL‐17RC, IL‐17RD, and IL‐17RE in healthy controls (HC; *n* = 20). (D) MFI of IL‐17RA, IL‐17RC, and IL‐17RD in HC (*n* = 20 for complete MFI measurements). Data are presented as medians and interquartile ranges, and each symbol represents one independent donor. Global within‐subject differences among IL‐17 receptor family members were assessed using the Friedman test. Pairwise post hoc comparisons were performed using the Wilcoxon signed‐rank test with Holm adjustment for multiple comparisons. The *p* values shown above the brackets correspond to Holm‐adjusted pairwise comparisons, whereas the upper horizontal arrow represents the global Friedman test (*p* < 0.0001 in all panels). Due to their low frequency of positive cells, IL‐17RB and IL‐17RE were not included in the MFI analysis.

Due to the very low frequencies of IL‐17RB‐ and IL‐17RE‐positive cells, MFI analyses were restricted to IL‐17RA, IL‐17RC, and IL‐17RD. The fluorescence measurements obtained of these receptors differed significantly within RA samples (*p* < 0.0001). The median MFI values were 9685 [IQR 8642.25–12,982.50] for IL‐17RC, 2236 [IQR 2141.00–2314.50] for IL‐17RA, and 1188 [IQR 1150.25–1261] for IL‐17RD (Figure 3B).

A similar receptor hierarchy was observed in HC, with significant differences among IL‐17R family members (*p* < 0.0001). The most expressed receptor was IL‐17RD with 14.40% (IQR 11.15–16.95), followed by IL‐17RA with 3.85% (IQR 2.49–4.77) and IL‐17RC with 0.40% (IQR 0.19–2.11). In contrast, IL‐17RB and IL‐17RE remained low (Figure 3C). MFI measurements for IL‐17RA, IL‐17RC, and IL‐17RD also differed significantly within HC (*p* < 0.0001), with median values of 2309 (IQR 2237.75–2435), 9564 (IQR 8573–10,592), and 1258 (IQR 1135.25–1354.75), respectively (Figure 3D).

### 3.4. Between‐Group Comparisons of IL‐17RA, IL‐17RC, and IL‐17RD

The percentage of IL‐17RA^+^ B cells was lower in RA patients than in HC (HC: 3.85% [IQR 2.49–4.77] vs. RA: 2.61% [IQR 1.80–3.44]; *p* = 0.0021), and this difference remained statistically significant after adjustment for multiple comparisons (*p* = 0.0126) (Figure 4A; Table 2).

**Figure 4:**
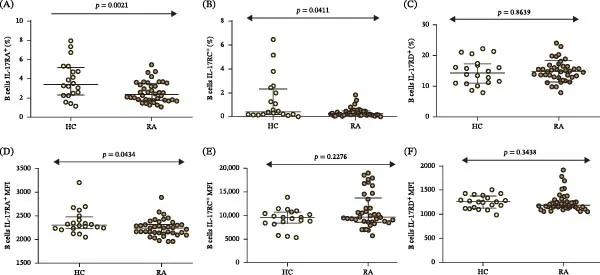
Differential expression of IL‐17 receptor family members on peripheral B lymphocytes in patients with rheumatoid arthritis and healthy controls. Comparison between patients with rheumatoid arthritis and healthy controls for the percentage of receptor‐positive CD19^+^ B cells (A, C) and mean fluorescence intensity (MFI) (D–F). (A–D) IL‐17RA; (B, E) IL‐17RC; (C–F) IL‐17RD. Data are presented as medians with interquartile ranges, and each symbol represents one independent donor. Each sample was stained and acquired once, with no technical replicates. Statistical comparisons between RA and HC were performed using the Mann–Whitney *U* test. The *p* values shown in the figure correspond to nominal comparisons; Holm‐adjusted *p* values for multiple comparisons are reported in Table 2.

**Table 2:** Expression of IL‐17 receptor family members on peripheral B lymphocytes.

Receptor	HC % positive	RA % positive	Nominal *p*	Holm‐adjusted *p*	HC MFI	RA MFI	Nominal *p*	Holm‐adjusted *p*
IL‐17RA	3.85 (2.49–4.77)	2.61 (1.80–3.44)	0.0021	0.0126	2309 (2237.75–2435)	2236 (2141–2314.5)	0.0434	0.2055
IL‐17RC	0.40 (0.19–2.11)	0.225 (0.13–0.48)	0.0411	0.2055	9564 (8573–10,592)	9685 (8642–12,982.5)	0.2276	0.6828
IL‐17RD	14.40 (11.15–16.95)	14.80 (12.90–16.40)	0.8639	0.8639	1258 (1135.25–1354.75)	1188 (1150.25–1261)	0.3438	0.6876

*Note*: Values are presented as median (interquartile range). Comparisons were performed using the Mann–Whitney *U* test. Nominal *p* values were adjusted for multiple comparisons using the Holm method.

The percentage of IL‐17RC^+^ B cells was also nominally lower in RA patients (HC: 0.40% [IQR 0.19–2.11] vs. RA: 0.225% [IQR 0.13–0.48]; *p* = 0.0411); however, this difference did not remain statistically significant after Holm adjustment (*p* = 0.2055) (Figure 4B; Table 2). No difference was observed in IL‐17RD‐positive B‐cell frequency (*p* = 0.8639; Holm‐adjusted *p* = 0.8639) (Figure 4C).

For MFI measurements, the nominal difference observed for IL‐17RA (HC: 2309 [IQR 2237.75–2435] vs. RA: 2236 [IQR 2141–2314.5]; *p* = 0.0434) did not remain significant after correction for multiple comparisons (Holm‐adjusted *p* = 0.2055) (Figure 4D). No significant between‐group differences were observed for IL‐17RC MFI (nominal *p* = 0.2276; Holm‐adjusted *p* = 0.6828) or IL‐17RD MFI (*p* = 0.3438; Holm‐adjusted *p* = 0.6876) (Figure 4E,F; Table 2).

### 3.5. IL‐17R Expression According to Clinical Activity (DAS28‐ESR)

RA patients were stratified according to DAS28‐ESR into remission (*n* = 10), low (*n* = 10), moderate (*n* = 12), and high disease activity (*n* = 10). The frequency of B cells did not differ among disease activity categories (*p* = 0.9076). Similarly, no significant differences were observed in either the percentage of receptor‐positive cells or MFI for IL‐17RA (*p* = 0.1728 and *p* = 0.7038, respectively), IL‐17RC (*p* = 0.9846and *p* = 0.3745, respectively), or IL‐17RD (*p* = 0.3075 and *p* = 0.1476, respectively) (Table 3).

**Table 3:** IL‐17 receptor expression on B lymphocytes stratified by disease activity.

Receptor	Measure	Remission (*n* = 10)	Low activity (*n* = 10)	Moderate activity (*n* = 12)	High activity (*n* = 10)	*p* value
B cells	%	7.64 (6.73–10.2)	6.89 (6.39–9.75)	6.7 (5.4–8.53)	6.61 (3.74–9.17)	0.9076
	MFI	2594.5 (2386.25–2701)	2434.5 (2094.75–2632)	2650 (2313.5–2863)	2346 (2062–2538)	0.4590
IL‐17RA	%	1.85 (1.71–2.13)	2.84 (1.93–3.65)	2.54 (1.75–3.20)	3.08 (2.13–3.58)	0.1728
	MFI	2216.5 (2047–2367)	2275 (2186.25–2417.25)	2252 (2164–2314.50)	2156.5 (2144.25–2329.75)	0.7038
IL‐17RC	%	0.33 (0.10–0.58)	0.21 (0.11–0.41)	0.24 (0.15–0.38)	0.24 (0.15–0.42)	0.9846
	MFI	9267.5 (8368–10621.25)	9661.5 (8636.25–11,184)	13,452 (9488.5–17,210)	10,307 (9275.25–15,728.25)	0.3745
IL‐17RD	%	13.65 (10.6–14.5)	15.95 (14.08–17.8)	15.00 (13.1–15.5)	14.85 (13.8–16.25)	0.3075
	MFI	1129.5 (1093.25–1161.5)	1195 (1183–1241)	1168 (1152–1219)	1207 (1170–1297)	0.1476

*Note*: Values are presented as median (interquartile range). Comparisons among disease activity groups were performed using the Kruskal–Wallis test. No post hoc pairwise comparisons were performed because none of the global comparisons reached statistical significance.

Abbreviation: MFI, mean fluorescence intensity.

## 4. Discussion

The present study provides a comparative characterization of the surface expression profile of IL‐17R family members on peripheral CD19^+^ B cells from patients with RA and HC. The frequency of receptor‐positive B cells differed markedly among IL‐17R family members within both groups, with IL‐17RD showing the highest proportion of positive cells, followed by IL‐17RA, whereas IL‐17RC was detected at lower frequencies, and IL‐17RB and IL‐17RE were infrequent. In between‐group comparisons, the proportion of IL‐17RA^+^ B cells was lower in patients with RA and remained statistically significant after adjustment for multiple comparisons. In contrast, the nominal difference observed for IL‐17RC^+^ B‐cell frequency did not remain significant after Holm adjustment, while IL‐17RD showed no differences between RA and HC. No significant differences in receptor expression were observed across DAS28‐ESR disease activity categories.

Beyond the formation of autoantibodies, B cells are widely acknowledged as key players in the pathophysiology of RA, playing a role in immune regulation, cytokine secretion, and antigen presentation [4–6, 22]. In this context, the lower proportion of circulating IL‐17RA^+^ B cells observed in RA is of interest because IL‐17RA participates in several IL‐17R complexes, including the IL‐17RA/IL‐17RC heterodimer involved in IL‐17A and IL‐17F signaling [12, 23, 24]. However, the present data demonstrate only a difference in the proportion of B cells with detectable surface IL‐17RA and do not establish the underlying mechanism or its functional consequences. Cytokine‐receptor surface availability can be influenced by processes such as receptor internalization, trafficking, glycosylation, and intracellular signaling pathways [25–28]. Therefore, the decreased percentage of IL‐17RA^+^ cells observed in RA might be an adaptive response aimed at preventing excessive IL‐17–mediated activation.

IL‐17RC deserves a similarly cautious interpretation. Although a lower proportion of IL‐17RC^+^ B cells was observed in RA at the nominal significance level, this association did not remain significant after adjustment for multiple comparisons. Accordingly, this finding should be considered exploratory rather than evidence of a disease‐associated alteration in IL‐17RC expression. Likewise, the between‐group differences initially observed for the IL‐17RA MFI did not remain significant after adjustment. Furthermore, raw MFI values obtained using different antibody–fluorochrome conjugates cannot be directly compared as quantitative estimates of receptor abundance. Thus, although fluorescence measurements differed among receptor‐specific conjugates within the study, these values should not be interpreted as demonstrating a greater receptor density for one IL‐17R family member relative to another.

IL‐17RD showed similar frequencies and MFI values in RA and HC. IL‐17RD has been described as a modulator of IL‐17 signaling and NF‐κB activation [29]; consequently, despite persistent systemic inflammation, its sustained expression in RA may indicate a maintained homeostatic control mechanism. The idea that receptor remodeling in RA is selective rather than universal across the IL‐17R family is supported by the receptors’ stability.

Circulating B cells had very low expressions of IL‐17RB and IL‐17RE, which is consistent with reports suggesting that they are confined in their tissue distribution, especially in mucosal and epithelial compartments [30, 31]. They are unlikely to play a major role in systemic B‐cell‐mediated autoimmunity given their low prevalence, although context‐dependent roles cannot be ruled out.

We did not find receptor expression changes among the disease activity groups. This observation raises the possibility that IL‐17R remodeling is a comparatively stable immunophenotypic characteristic rather than a dynamic marker of a fluctuating inflammatory state. However, the cross‐sectional design restricts inferences about treatment effects or changes over time. It will be necessary to conduct longitudinal research that includes functional measurements and synovial tissue analysis to determine whether receptor expression varies as the disease progresses or with treatment [32].

These results affect how the moderate clinical effectiveness of IL‐17‐targeted treatments for RA is interpreted. Response to treatment may be influenced by receptor expression in particular immune compartments [18, 19]. Reduced IL‐17RA and IL‐17RC expression on B cells could limit the impact of receptor‐directed blockade in this population, highlighting the importance of cell‐type‐specific receptor landscapes when designing immunomodulatory strategies. A more accurate method of modifying IL‐17‐driven pathways would be to selectively target receptor combinations rather than global cytokine inhibition [33, 34].

Several limitations need to be acknowledged. First, this was a cross‐sectional study, preventing conclusions regarding temporal or causal relationships between RA and IL‐17R surface expression. Second, surface receptor expression does not necessarily reflect downstream signaling capacity, and we did not assess receptor internalization, posttranslational modification, or intracellular signaling events [26, 27, 35]. Furthermore, because B‐cell activation is more prominent in the synovial tissue of RA patients, peripheral blood may not adequately reflect the immunological processes occurring in the synovial tissue. Finally, all participants were women. Although the inclusion of women was defined a priori to reduce sex‐related biological variability within the study population and reflects the higher prevalence of RA among women, the present findings cannot be generalized to male patients and should be validated in cohorts including both sexes. Despite these constraints, the current study offers the first thorough characterization of the IL‐17R family in human peripheral B cells in RA.

In conclusion, the present findings identify a distinct surface‐expression profile of IL‐17R family members on peripheral B cells and demonstrate a lower proportion of IL‐17RA^+^ B cells in RA that remained significant after correction for multiple comparisons. The nominal difference observed for IL‐17RC did not remain significant after adjustment, whereas IL‐17RD expression was comparable between RA and HC. These observations provide a descriptive basis for further investigation of IL‐17R biology in human B cells [9, 11, 12]. Future research should clarify how these phenotypic alterations affect B‐cell function and signaling, particularly within the synovial microenvironment.

## Author Contributions

**Coronado González Christian Fernando**: conceptualization, methodology, software, formal analysis, investigation, data curation, writing – original draft, visualization, supervision. **Sifuentes Franco Sonia**: conceptualization, methodology, software, visualization, writing – original draft, supervision. **Hernández Bello Jorge and Muñoz Valle José Francisco**: methodology, software, formal analysis, investigation, writing – original draft. **Graciano Machuca Omar**: methodology, software, visualization, supervision. **López Pérez Iván**: methodology, software, visualization, supervision. **Morales Núñez José Javier**: conceptualization, methodology, resources, writing – original draft, visualization, supervision, funding acquisition.

## Funding

This research was funded by the PROGRAMA DE FOMENTO A LA INVESTIGACIÓN 2023–2024 program of the University of Guadalajara, Centro Universitario de los Valles.

## Ethics Statement

The Ethics Committee of the “Centro Universitario de los Valles” of the University of Guadalajara (Protocol Code CI‐CEI/47/2023) approved this study, which was conducted according to the guidelines of the Declaration of Helsinki. Written informed consent was obtained from all the patients to publish this article.

## Conflicts of Interest

The authors declare no conflicts of interest.

## Supporting Information

Additional supporting information can be found online in the Supporting Information section.

## Supporting information


**Supporting Information** Figure S1: Fluorescence‐minus‐one controls used to define IL‐17 receptor‐positive B‐cell populations. Representative fluorescence‐minus‐one (FMO) controls were used to establish the positivity threshold for each member of the IL‐17 receptor family (IL‐17RA, IL‐17RB, IL‐17RC, IL‐17RD, and IL‐17RE). The same sequential gating strategy used for the study samples was applied: lymphocytes were selected according to FSC‐A and SSC‐A characteristics, singlets were identified using FSC‐A and FSC‐H, and CD19⁺ B cells were subsequently gated. For each receptor‐specific FMO control, the corresponding antibody was omitted while the remaining antibodies in the panel were retained. The receptor‐positive gate was established at the fluorescence boundary defined by its corresponding FMO distribution, and events located beyond this boundary were considered positive in fully stained samples. Unstained cells were additionally used to assess background autofluorescence and confirm the absence of detectable fluorescence in the corresponding channels. Table S1: Event counts for the representative flow‐cytometry gating strategy.

## Data Availability

The data that support the findings of this study are available from the corresponding author upon reasonable request.
