# Supplementary material for: Expression Profile of the IL‐17 Receptor Family on Peripheral B Lymphocytes in Rheumatoid Arthritis
Source: J Immunol Res. 2026 Oct 23;2026:5266857. doi: 10.1155/jimr/5266857 (PMC13691522; doi:10.1155/jimr/5266857)
Supplement: Supplementary file 1 [file JIMR-2026-5266857-s001.docx]

Supplementary Figure 1. Fluorescence-minus-one controls used to define IL-17 receptor-positive B-cell populations**.** Representative fluorescence-minus-one (FMO) controls were used to establish the positivity threshold for each member of the IL-17 receptor family (IL-17RA, IL-17RB, IL-17RC, IL-17RD, and IL-17RE). The same sequential gating strategy used for the study samples was applied: lymphocytes were selected according to FSC-A and SSC-A characteristics, singlets were identified using FSC-A and FSC-H, and CD19⁺ B cells were subsequently gated. For each receptor-specific FMO control, the corresponding antibody was omitted while the remaining antibodies in the panel were retained. The receptor-positive gate was established at the fluorescence boundary defined by its corresponding FMO distribution, and events located beyond this boundary were considered positive in fully stained samples. Unstained cells were additionally used to assess background autofluorescence and confirm the absence of detectable fluorescence in the corresponding channels

**Supplementary Table 1. Event counts for the representative flow-cytometry gating strategy**

| Representative RA and healthy-control samples | | | | | |
| --- | --- | --- | --- | --- | --- |
| Population | **Healthy control, n** | **Healthy control, % of parent** | | **RA patient, n** | **RA patient, % of parent** |
| Total acquired events | 215,143 | — | | 274,783 | — |
| Lymphocytes | 86,509 | 40.2 | | 50,251 | 18.3 |
| Singlets | 82,381 | 95.2 | | 49,092 | 97.7 |
| CD19⁺ B cells | 4,322 | 5.25 | | 2,628 | 5.35 |
| IL-17RA⁺ B cells | 203 | 4.70 | | 51 | 1.94 |
| IL-17RB⁺ B cells | 17 | 0.39 | | 0 | 0 |
| IL-17RC⁺ B cells | 226 | 5.23 | | 1 | 0.038 |
| IL-17RD⁺ B cells | 471 | 10.9 | | 362 | 13.8 |
| IL-17RE⁺ B cells | 15 | 0.35 | | 3 | 0.11 |
| FMO background levels | | | | | |
| FMO control | **Corresponding positive gate** | | **Events in positive region, n** | | **Background, %** |
| FMO IL-17RA | IL-17RA⁺ | | 0 | | 0 |
| FMO IL-17RB | IL-17RB⁺ | | 8 | | 0.27 |
| FMO IL-17RC | IL-17RC⁺ | | 2 | | 0.077 |
| FMO IL-17RD | IL-17RD⁺ | | 4 | | 0.12 |
| FMO IL-17RE | IL-17RE⁺ | | 0 | | 0 |

*Event counts correspond to the representative healthy-control and rheumatoid arthritis samples shown in Figure 1. Percentages for lymphocytes, singlets, and CD19⁺ B cells are expressed relative to their respective parent populations. Percentages of IL-17 receptor-positive cells are expressed relative to the CD19⁺ B-cell population. FMO background represents events falling within the corresponding receptor-positive gate when that specific antibody was omitted.*
